# On the Impact of Multiple Access Interference in LTE-V2X and NR-V2X Sidelink Communications †

**DOI:** 10.3390/s23104901

**Published:** 2023-05-19

**Authors:** Abdul Rehman, Roberto Valentini, Elena Cinque, Piergiuseppe Di Marco, Fortunato Santucci

**Affiliations:** 1 Center of Excellence EX-EMERGE, Department of Information Engineering, Computer Science and Mathematics, University of L’Aquila, 67100 L’Aquila, Italy; abdul.rehman@graduate.univaq.it (A.R.); roberto.valentini@univaq.it (R.V.); elena.cinque@univaq.it (E.C.); fortunato.santucci@univaq.it (F.S.); 2 Radiolabs Consortium, 00198 Roma, Italy

**Keywords:** 5G NR-V2X, LTE-V2X, C-V2X, Mode 2, Mode 4, 3GPP, analytical model, SINR, BLER, vehicular communications, multiple access interference, platooning

## Abstract

Developing radio access technologies that enable reliable and low-latency vehicular communications have become of the utmost importance with the rise of interest in autonomous vehicles. The Third Generation Partnership Project (3GPP) has developed Vehicle to Everything (V2X) specifications based on the 5G New Radio Air Interface (NR-V2X) to support connected and automated driving use cases, with strict requirements to fulfill the constantly evolving vehicular applications, communication, and service demands of connected vehicles, such as ultra-low latency and ultra-high reliability. This paper presents an analytical model for evaluating the performance of NR-V2X communications, with particular reference to the sensing-based semi-persistent scheduling operation defined in the NR-V2X Mode 2, in comparison with legacy sidelink V2X over LTE, specified as LTE-V2X Mode 4. We consider a vehicle platooning scenario and evaluate the impact of multiple access interference on the packet success probability, by varying the available resources, the number of interfering vehicles, and their relative positions. The average packet success probability is determined analytically for LTE-V2X and NR-V2X, taking into account the different physical layer specifications, and the Moment Matching Approximation (MMA) is used to approximate the statistics of the signal-to-interference-plus-noise ratio (SINR) under the assumption of a Nakagami-lognormal composite channel model. The analytical approximation is validated against extensive Matlab simulations that a show good accuracy. The results confirm a boost in performance with NR-V2X against LTE-V2X, particularly for high inter-vehicle distance and a large number of vehicles, providing a concise yet accurate modeling rationale for planning and adaptation of the configuration and parameter setup of vehicle platoons, without having to resort to extensive computer simulation or experimental measurements.

## 1. Introduction

Vehicular communications are envisioned as a major enabler to allow intelligent transportation systems (ITS) and smart cities to become completely integrated, especially in very densely populated places. The transition to a cellular radio access infrastructure is planned as a strategic necessity to develop automated driving and its integration into the larger transport ecosystem [1], supported by all the involved actors (i.e., network operators and vehicle manufacturers). From this perspective, vehicular communications have advantages beyond just safety applications. Vehicles with vehicle-to-everything (V2X) connectivity can aid in improving traffic management, which results in greener automobiles and cheaper fuel costs [2].

The vehicle safety and non-safety applications lay the foundation for Cooperative-Intelligent Transportation Systems (C-ITSs), which the European Commission has been promoting [3], as well as Cooperative, Connected, Automated, and Autonomous Mobility (CCAM) [4]. C-ITS services were given 75 MHz of the spectrum in the 5.9 GHz band by the US Federal Communications Commission (FCC) in 1999. Throughout the past 20 years, the allocation has spurred a great deal of research to design and implement V2X communications (e.g., the CAMP collaboration in the US [5], the Car 2 Car Communication Consortium in Europe, [6] and many more research initiatives).

Subsequently, starting with Release 14 (Rel-14), the 3rd Generation Partnership Project (3GPP) concentrated on developing the cellular V2X technology, establishing several specification standards to advance the V2X technology. In this domain, different communication modes can be identified, e.g., Vehicle-to-Vehicle (V2V), Vehicle-to-Network (V2N) or Infrastructure (V2I), Vehicle-to-Road Side Unit (V2R), and Vehicle-to-Pedestrian (V2P). The definition of higher layer standards, message formats, protocols, and applications (such as [7] in Europe and [8] in the US) came after the development of the first IEEE 802.11-based radio standards [9]. Cross-domain alliances have been established as a tangible result, such as the 5G Automotive Association (5GAA), which is very active in defining development plans and use cases for various service classes as well as in stating precise requirements (such as E2E latency, bit rates, availability, and loss rates) for standardization bodies such as the 3GPP [10].

Most challenging use cases, e.g., vehicle platooning or cooperative collision avoidance (CCA), present difficulties and opportunities for Cellular-V2X (C-V2X) communications [11]. Additionally, due to the stringent latency requirement (even below 20 ms [10]), direct radio communication between vehicles, i.e., sidelink communication, is preferable to allocating resources through the cellular infrastructure. However, the achievable performance is limited by multiple access interference in C-V2X sidelink communication, particularly in congested metropolitan environments. Additionally, 3GPP Rel-14 Mode 3 and Mode 4 are sometimes called the baseline modes for sidelink LTE-V2X [12]. The Semi-persistent Scheduling (SPS) scheme, exploited for resource allocation, may not be able to deliver the necessary level of service depending on the distances and relative positions between vehicles [13]. It is crucial to characterize the performance of such systems analytically, to plan and manage the settings for vehicle platooning.

With Release 15 (Rel-15), the 3GPP started a new cellular V2X standard based on the 5G New Radio (NR) air interface. Then, based on the 5G NR air interface, Release 16 (Rel-16) is the first to enable NR-V2X sidelink communication, and serves as the foundation for upcoming improvements and extensions for both NR-V2X and non-V2X sidelink applications [14]. From the start of the work on NR-V2X, 3GPP decided that NR-V2X needed to complement LTE-V2X and not replace it [15]. LTE-V2X is envisioned to support basic active safety applications, whereas NR-V2X will support more advanced applications, including connected and automated driving. Supporting enhancements to V2X (eV2X) use cases associated with connected and autonomous driving is the aim of the NR-V2X sideline. The counterparts in NR-V2X of LTE-V2X Mode 3 and Mode 4 are NR-V2X Mode 1, which utilizes an altered version of the sensing-based SPS (SB-SPS) algorithm, for planned resource selection, and NR-V2X Mode 2, which employs autonomous resource selection.

Sehla et al. [16] discussed the main differences, challenges, potential technical developments, and open issues related to resource allocation in C-V2X technologies. The performance of C-V2X systems has been predominantly examined in the literature via simulations [17,18]. Most analytical models consider IEEE 802.11-based V2X [19] or LTE-V2X Mode 3 [20] with infrastructure support. For LTE-V2X communications, Mode 3 and Mode 4 were developed to satisfy latency requirements while accommodating significant Doppler spreads and vehicle density. The research in [21] proposed a model that measures the four different types of packet errors that affect the performance of LTE-V2X sidelink: errors caused by half-duplex transmissions, errors caused by received signal power below the sensing power threshold, errors caused by propagation effects, and errors caused by packet collisions. The multiple access interference scenario is modeled using simplified assumptions, even though the model is quite accurate in its depiction of the SPS scheme. As an example, a random traffic generation pattern is the way to define the presence of automobiles statistically.

In our previous work in [22], we developed an analytical framework for the LTE-V2X sidelink communication protocol in terms of packet success probability by simulating the intricate relationship between the SB-SPS and the propagation environment. In this paper, we extend the analysis to include the relevant characteristics of NR-V2X communications. Since this distribution fits in more measurement campaigns than the other distributions, the Nakagami-lognormal distribution is often employed for model fading. This distribution was first published in [23] to evaluate the outage probability in scenarios with several co-channel interferers. The difficulty in differentiating between small-scale fading and shadowing processes in compact stationary contexts such as automotive environments and vehicular communication has recently increased interest in this distribution [24].

We first derive an approximation of the probability density function (PDF) for the signal-to-interference-and-noise ratio (SINR) by using the Nakagami-lognormal propagation model and multiple-access interference. To achieve this, the SINR is approximated via a lognormal random variable, the statistics of which are acquired by moment matching [25,26,27]. This approach is more convenient than stochastic geometry (e.g., as in [28]) since the performance needs to be derived for the specific relative distance between vehicles and it is important to be able to distinguish the SINR for a specific vehicle in transmission with interferers at a relatively fixed position, as in platooning scenarios. Finally, using the Look-Up Table (LUT) reasoning outlined in [29], the packet success probability is determined numerically. The SB-SPS technique is explicitly accounted for to characterize the interference patterns, and the resulting channel schedule is used to derive the SINR statistics. Our model, which is specifically tailored to the platooning scenario, allows us to describe the packet success probability as a function of the number of interfering vehicles, the number of available resources, and the distance between the transmitter and the receiver. This contrasts with earlier works in the literature (e.g., [21]). The proposed approach also captures the intricate interplay between the channel access strategy and the propagation environment. Extensive Monte Carlo (MC) simulations are used to validate the proposed analytical model’s performance predictions and approximation theory, and it is shown that there is good agreement between the approximated and simulated results for both LTE-V2X Mode 4 and NR-V2X Mode 2. In summary, the main contributions of this paper are the following.

- A vehicle platooning use case scenario is illustrated and analyzed by considering the evolution of the standards from LTE-V2X to 5G NR-V2X sidelink operations.
- An analytical model of the multiple access interference due to the sensing-based semi-persistent scheduling operation, based on the preliminary work on LTE-V2X in [22], is provided and validated for the considered scenario.
- A framework for comparison of the successful packet delivery performance of two technologies is provided, accounting for the scheduling of available resources, the number of interfering vehicles, and their relative positions.
- As an interesting insight, the interplay between an improved sensing-based scheduled access and the enhanced physical layer specification in 5G NR-V2X Mode 2 is shown to be particularly beneficial for the performance as the number of vehicles and the inter-vehicle distance increase.

The rest of this paper is organized as follows: The system model is presented in Section 2. The Cellular-V2X technologies and the analytical characterization of the sidelink operations are described in Section 3 and Section 4, respectively. The comparison of LTE-V2X Mode 4 and NR-V2X Mode 2 is validated by the proposed analytical model and Monte Carlo simulations in Results Section 5. Finally, Section 6 concludes the paper.

## 2. System Model

Driving safety, traffic quality, and infotainment features are all significantly improved by V2X, which enables vehicular nodes to acquire a wide variety of traffic data in real-time. A large amount of data, including cooperative sensing data, communications, entertainment, sensing data, etc., is produced as vehicles become more advanced and autonomous due to the usage of numerous sensors and a variety of communication devices and technologies. A detailed explanation of such V2X use cases and specifications can be found in [30]. Additionally, 3GPP specifies various levels of automation for each use case, ranging from 0 (no automation) to 5 (full automation), in compliance with the definition by the Society of Automotive Engineers (SAE). Vehicle platooning use cases with the dynamic formation and administration of groups of vehicles in platoons are envisioned by 3GPP. For the platoon to operate properly, vehicles in it periodically exchange data. The available QoS may affect how far apart each vehicle in a platoon travels.

**Figure 1:**
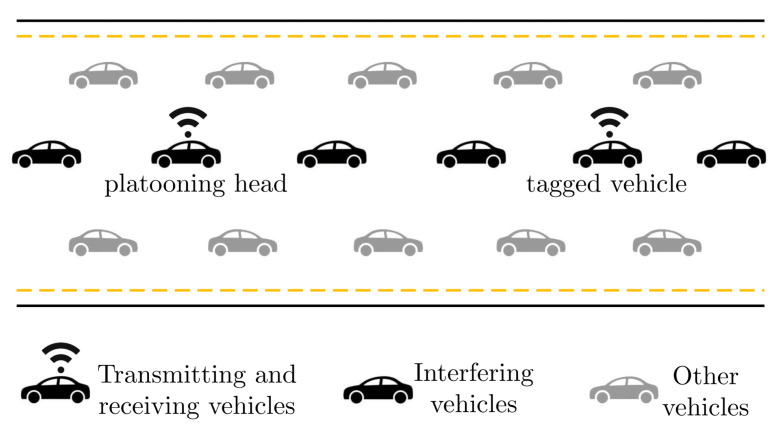
Considered vehicle platooning scenario.

A vehicle platooning scenario with a set of V2X-enabled vehicles and a set of non-communicating vehicles is considered in this work. Without a loss of generality, we refer to the communication between a tagged transmitting vehicle and the platoon head, which acts as the tagged receiver, as shown in Figure 1. The distance between communication entities is constant, and we adjust the parameters by the specified vehicle platooning use case [30]. According to 3GPP, the scenario under consideration calls for up to 100 messages per second, a maximum speed of 100 km/h, a minimum distance between vehicles of a few meters, and message sizes ranging from roughly 50 to 1200 bytes with a 90% reliability.

We consider the LTE-V2X and NR-V2X physical layer specifications and the V2X sidelink access protocol based on SB-SPS to control access to the shared resources, which are illustrated in Section 4. Lastly, all packets are transmitted using the same modulation and coding scheme (MCS), and all packets are of the same size.

## 3. Cellular-V2X Technologies

In this section, we review the key technological aspects related to the evolution of V2X systems towards 5G systems [31], focusing on the transmission modes for sidelink operations in LTE-V2X and NR-V2X.

### 3.1. LTE-V2X

The C-V2X was initially created by the 3GPP based on the V2X specifications in Rel-14 as LTE-V2X (also named LTE-V), which gives top priority to radio access improvements appropriate for V2X. Until then, IEEE 802.11-based solutions mainly covered V2X scenarios. By employing already-existing cell towers, the LTE-V2X promised minimal costs, quick development, and installation to improve the accessibility and efficiency of the public transportation system. LTE-V2X can use a licensed cellular network for commercial purposes such as voice or data access. Moreover, LTE-V2X enables sidelink direct V2V connections via the so-called PC5 interface. The vehicular nodes communicate with other nodes directly over the same channel at the PC5, with no intervention from the infrastructure.

LTE-V2X uses the 5.9 GHz band, which has been reserved in several regions (such as the United States, Europe, and China) for ITS services. LTE-V2X supports cooperative active traffic safety, traffic management, and telematics applications. Similar services are supported by LTE-V2X as they are by IEEE 802.11-based V2X or its equivalent, ETSI ITS-G5. These services depend on the broadcast transmission of brief awareness messages called CAMs (Cooperative Awareness Messages) in ETSI ITS-G5 [7] or SAE BSMs (Basic Safety Messages) in IEEE 802.11 V2X [32] to provide essential data such as the location, direction, speed, and acceleration of the transmitting vehicle regularly. LTE-V2X reuses the higher V2X layers and protocols provided by ETSI and SAE while defining new Physical (PHY) and Medium Access Control (MAC) layers for V2X.

Sidelink LTE-V2X supports 10 and 20 MHz channels in the 5.9 GHz band. At the physical and medium access control (MAC) levels, single-carrier frequency-division multiple access (SC-FDMA) is used. The channel is divided into resource blocks (RBs) that occupy the 180 kHz bandwidth with 12 sub-carriers. In the time domain, the duration of each RB is 1 ms, which is the minimum allocation time (MAT) and can carry 14 orthogonal frequency-division multiplexing (OFDM) symbols. Nine symbols carry data, and four symbols are reserved for channel estimation. One symbol is used to adjust the time and may also indicate a switch between the transmitter and receiver. RBs can be grouped into sub-channels of variable size, and a data packet is carried over sub-channels in a transport block (TB).

As mentioned above, to accommodate low latency V2X communications, 3GPP Rel-14 introduced two specific transmission modes (Modes 3 and 4); see [12]. Vehicles can employ LTE-V2X Mode 4, which allows vehicles to autonomously reserve resources using the resource reservation algorithm, while they are outside cellular coverage. The standard defines a sensing-based scheduling approach for resource allocation among neighboring vehicles. The time frame in which resources are reserved by a vehicle is known as the resource reservation interval (RRI). LTE-V2X Mode 4 uses RRI values of 0, 20, 50 ms, and multiples of 100 ms, up to 1 s. The resource reservation algorithm requires each vehicle to sense the channel for 1 s to identify candidate resources to be excluded, therefore decreasing packet collisions.

### 3.2. NR-V2X

The NR-V2X is built to handle V2X applications with a range of latency, reliability, and throughput requirements. Rel-16 includes two modes for selecting sub-channels in NR-V2X sidelink communications using the NR-V2X PC5 interface (Mode 1 and Mode 2). These two modes replace Mode 3 and Mode 4 of LTE-V2X, respectively. Nevertheless, LTE-V2X only enables broadcast communications in the sidelink, while NR-V2X supports broadcast, groupcast, and unicast sidelink communications.

Similar to LTE-V2X Mode 4, UEs can autonomously select their sidelink resources (one, or several sub-channels) from a resource pool when using NR-V2X Mode 2. In both cases, the resource pool can be preconfigured by the infrastructure with a list of approved RRIs. There can be a maximum of 16 separate RRIs on the list. LTE-V2X Mode 2 and NR-V2X Mode 4 have some minor differences in the scheduling scheme, in particular, when related to retransmissions. Mode 4 operates following a semi-persistent scheduling scheme. On the other hand, Mode 2 can operate using both a dynamic and a semi-persistent scheduling scheme. The dynamic scheme selects new resources for each TB and can only reserve resources for the retransmissions of that TB. The RRI determines the interval between the resources chosen for the transmission of consecutive TBs for NR-V2X. NR-V2X Mode 4 has more flexibility in the selection of the RRI compared to LTE-V2X, by allowing any integer RRI between 1 and 99 ms. Finally, NR-V2X defines a lower constraint of 10 for the selection window value to guarantee a radio link layer latency of 10 ms [33]. Table 1 summarizes the key differences between the features/mechanisms of LTE-V2X and NR-V2X.

For Release 17 (Rel-17) [34], a new work item on the NR sidelink upgrade has been approved. This Rel-17 work item aims at improving Rel-16 NR-V2X resource allocation in Mode 2 in terms of power saving, dependability, and latency. Any improvements must be capable of coexisting with Rel-16 NR-V2X in the same resource pool (i.e., co-channel coexistence). Rel-16, NR-V2X Mode 1 and Mode 2 were created for UEs such as cars or remote sensor units (RSUs), which do not experience severe power constraints. Other UE kinds that are employed by pedestrians, and smartphones, also have the same constraints. A UE needs lengthy sensing intervals under the current Rel-16 Mode 2 requirements, which harms the battery life. A modification of Mode 2 that would use partial sensing to cut down on power consumption has been considered for investigation under Rel-17 [34].

## 4. Analytical Characterization of the Sidelink Operations

In this section, we provide an analytical characterization of the multiple access interference affecting C-V2X sidelink operations, referring to the system scenario provided in Section 2. The link between the tagged transmitter and the platooning head in Figure 1 is considered, and all the other communicating vehicles are treated as interfering vehicles. We denote the tagged vehicle as *i* and assume that its transmit power is $P_{\mathrm{tx},i}$. Thus, it is expected that both multi-path propagation and shadowing caused by impediments will negatively impact communication between the vehicle *i* and the platoon head vehicle *j*.

The received power at the platoon head can be expressed as follows by adopting an inverse power model for the link gain:

(1) $$\begin{array}{c} \begin{array}{c} P_{\mathrm{rx},i,j}=\frac{c_{0}P_{\mathrm{tx},i}}{d_{i,j}^{\alpha }}f_{i}\exp\left(y_{i}\right), \end{array} \end{array}$$

where $d_{i,j}$ is the distance between the sending and receiving vehicles. The constant $c_{0}=C_{0}G_{t}G_{r}$ includes the power gain at the reference distance of 1 m $C_{0}$, the transmit antenna gain $G_{t}$ and the receiving antenna gain $G_{r}$. In the operating conditions for V2X networks, the inverse of $c_{0}$ (i.e., the path loss at the reference distance) is in the range of 40–60 dB [35]. The path loss exponent α, ranges from 2 for open space propagation to 6 for severe propagation conditions [36]. Under the hypothesis of Nakagami distributed fading, the factor $f_{i}$ simulates the channel power gain due to multi-path propagation, which exhibits the Gamma distribution. We assume a Nakagami distribution with a parameter κ≥0.5 and p.d.f.

(2) $$\begin{array}{c} \begin{array}{c} p_{f_{i}}\left(z\right)=\kappa^{\kappa }\frac{{\left(z\right)}^{\kappa -1}}{\Gamma (\kappa )}\exp\left(-\kappa z\right), \end{array} \end{array}$$

where Γ(κ) is the fundamental Gamma function $\Gamma \left(\kappa \right)=\int_{0}^{\infty }\exp\left(-x\right)x^{\kappa -1}dx$. We point out that the Nakagami distribution can simulate fading environments with varying degrees of severity and is extremely general. Shadow fading is here modeled by the normal random variable $y_{i}∼N\left(0,\sigma_{i}^{2}\right)$, and thus the shadowing term $\exp(y_{i})$ in Equation (1) follows the log-normal distribution. The model we adopted is reliable for urban environments, in which devices may not be always in visibility. In a real-world system, the transmission power, path loss parameters, and standard deviation of the shadow fading of each interfering transmitting vehicle can vary and this needs to be considered in the model.

### 4.1. Sidelink Operations

**Figure 2:**
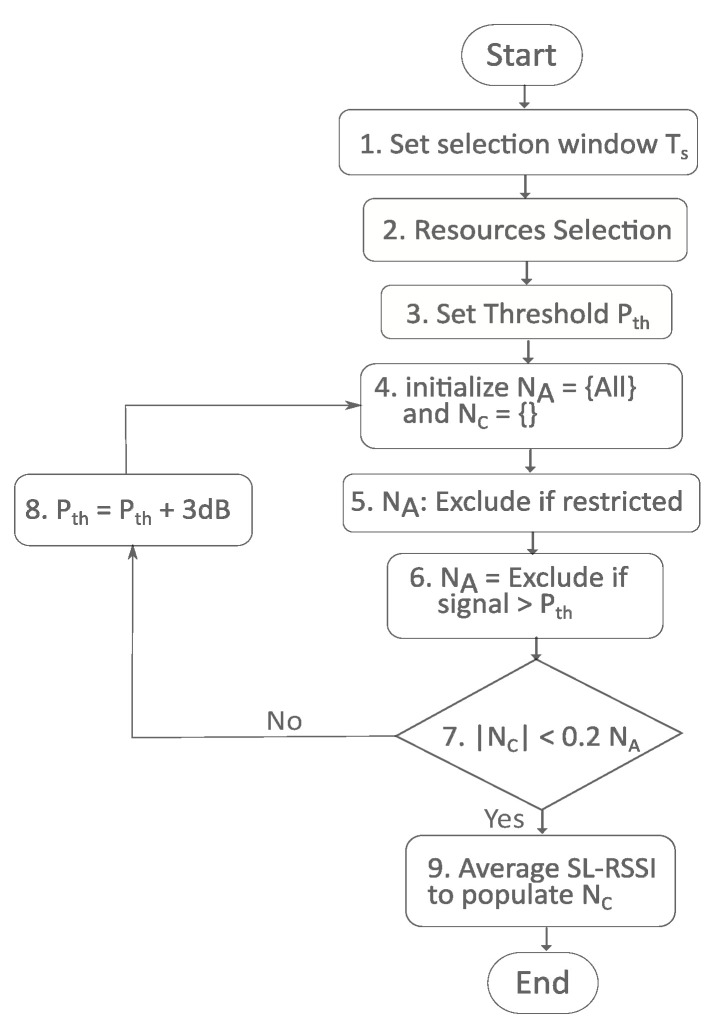
Semi-persistent scheduling in cellular V2X.

The detailed description in terms of physical and MAC layers and other parameters can be found in the previous Section 3. Here, we characterize the behavior of the underlying SB-SPS scheme of LTE-V2X Mode 4 and NR-V2X Mode 2. The fundamental objective of the SB-SPS algorithm is to identify a candidate group of sub-channels for the transmission of V2X. The resources are selected using channel sensing and then reserved for later use. Mode 2 allows for the possibility to dynamically re-select resources for retransmission. The general procedure of the SB-SPS system is illustrated in Figure 2.

The steps that each vehicle takes to find possible resources for transmission using SB-SPS are listed below.

- Channel sensing: the sidelink received signal strength indication (SL-RSSI) for each sub-channel is used to calculate the interference level for each sub-frame for each vehicle. This sensor information is obtained over a specific period (sensing time $T_{s}$).
- Selection of potential resources: every vehicle organizes its available resources ($N_{A}$) according to sensing measures, and the resources are chosen by the following rules: (i) the SL-RSSI of the selected resource must be lower than a predetermined threshold $P_{th}$ agreed by the vehicle); (ii) if the resource is not detected during $T_{s}$, it must not be chosen; (iii) out of all the resources accessible, no resources may be chosen that are already reserved; (iv) a vehicle cannot sense the channel when transmitting following a half-duplex transmission; and (v) the threshold $P_{th}$ is raised by 3 dB and the two-step exclusion process is repeated if the remaining candidate resources $N_{C}$ represent less than 20% of the total amount of resources $N_{A}$.
- Resource selection: the vehicle randomly chooses transmission resources from those it has identified as the best 20%. For an arbitrary number of successive transmissions with the same transmission interval, the vehicle may reserve the same resources. The SPS implementation of LTE-V2X and NR-V2X specifies the minimal and maximal number of successive transmissions before reallocation.
- Resources re-selection: each time a packet is transmitted, the SB-SPS counter, which shows how many more consecutive packets are left, is decreased. The vehicle can either keep the resources with a probability of $p_{k}$ or re-select the resources with a probability of $1-p_{k}$ when the counter reaches zero.

### 4.2. Model of Aggregate Fading Components

To determine the system’s performance in terms of packet success probability, the statistical characterization of the SINR is an essential step. The SINR associated to the tagged transmitter *i* and measured at the platoon head *j* is:

(3) $$\begin{array}{c} \begin{array}{c} SINR=\frac{P_{\mathrm{rx},i,j}}{\sum_{n\in I}P_{\mathrm{rx},n,j}+\sigma_{w}^{2}}=f_{i}{\left(\sum_{n\in I}\frac{P_{\mathrm{tx},n}d_{i,j}^{\alpha }}{P_{\mathrm{tx},i}d_{n,j}^{\alpha }}f_{n}\exp\left(y_{n}-y_{i}\right)+\frac{\sigma_{w}^{2}d_{i,j}^{\alpha }}{c_{0}P_{\mathrm{tx},i}}\exp\left(-y_{i}\right)\right)}^{-1}, \end{array} \end{array}$$

where $\sigma_{w}^{2}$ is the noise power and I is the set of interfering vehicles that select the same resource (i.e., the same sub-channel and sub-frame). The composition of I depends on the resource schedule provided by the SPS procedure. According to Equation (3) and given the power model of Equation (1), we can see that the PDF expression is not available in closed form when the SINR is defined as a weighted sum of log-normal random variables. As a result, we give an approximate description of the SINR data as follows:

Firstly, let us elaborate on the random variable:

(4) $$\begin{array}{c} {\tilde{Y}}_{i,j}=-\ln\left(\sum_{n\in \bar{I}}B_{i,j,n}\exp\left({\tilde{y}}_{n}\right)\right), \end{array}$$

where $\bar{I}=\left\{0;I\right\}$,

(5) $$\begin{array}{c} B_{i,j,n}=\left\{\begin{array}{cc} \begin{array}{c} \frac{\sigma_{w}^{2}d_{i,j}^{\alpha }}{c_{0}P_{\mathrm{tx},i}} \end{array} & n=0,\\ \begin{array}{c} \frac{P_{\mathrm{tx},n}d_{i,j}^{\alpha }}{P_{\mathrm{tx},i}d_{n,j}^{\alpha }}f_{n} \end{array} & n\in I, \end{array}\right. \end{array}$$

and

(6) $$\begin{array}{c} \tilde{y_{n}}=\left\{\begin{array}{cc} -y_{i} & n=0,\\ y_{n}-y_{i} & n\in I, \end{array}\right. \end{array}$$

Then, according to the moment matching approximation (MMA) method [25], we approximate the random variable in Equation (4) as ${\tilde{Y}}_{i,j}\approx Z$, where $Z∼N(\eta_{Z},\sigma_{Z}^{2})$. The unknown parameters $\eta_{Z}$ and $\sigma_{Z}^{2}$ can be obtained by matching the first two moments of exp(Z) with the first two moments of $\sum_{n=1}^{N}B_{i,j,n}\exp\left({\tilde{y}}_{n}\right)$. Formally,

(7) $$\begin{array}{cc} {\tilde{M}}_{1} & ≜\exp\left(-\eta_{Z}+\frac{1}{2}\sigma_{Z}^{2}\right)=\sum_{n\in \bar{I}}E\left\{B_{i,j,n}\right\}\exp\left(\frac{1}{2}\sigma_{{\tilde{y}}_{n}}\;\right), \end{array}$$

(8) $$\begin{array}{cc} {\tilde{M}}_{2} & ≜\exp\left(-2\eta_{Z}+2\sigma_{Z}^{2}\right)=\sum_{m\in \bar{I}}\sum_{n\in \bar{I}}E\left\{B_{i,j,m}B_{i,j,n}\right\}\times \exp\left(\frac{\sigma_{{\tilde{y}}_{m}}^{2}}{2}+\frac{\sigma_{{\tilde{y}}_{n}}^{2}}{2}+\rho_{{\tilde{y}}_{m},{\tilde{y}}_{n}}\sigma_{{\tilde{y}}_{m}}\sigma_{{\tilde{y}}_{n}}\right), \end{array}$$

where E{·} is the expectation operator, and $\rho_{{\tilde{y}}_{m},{\tilde{y}}_{n}}$ is the cross-correlation coefficient between two shadowing components. By solving the above system of equations, we obtain the unknown parameters:

(9) $$\begin{array}{c} \eta_{Z}=0.5\ln\left({\tilde{M}}_{2}\right)-2\ln\left({\tilde{M}}_{1}\right), \end{array}$$

and

(10) $$\begin{array}{c} \sigma_{Z}^{2}=\ln\left({\tilde{M}}_{2}\right)-2\ln\left({\tilde{M}}_{1}\right), \end{array}$$

Observe that ${\tilde{M}}_{1}$ and ${\tilde{M}}_{2}$ depend on the moment of $f_{i}$, which are given as $E\{f_{i}\}=1$ and $E\left\{f_{i}^{2}\right\}=\frac{\kappa +1}{\kappa }$ for Gamma distributed multi-path power gain. Once the unknown parameters are found, the SINR can be approximated as $SINR\approx \tilde{\gamma }$, where

(11) $$\begin{array}{c} \tilde{\gamma }=f_{i}\exp\left(Z\right), \end{array}$$

with $\tilde{\gamma }$ having p.d.f.

(12) $$\begin{array}{cc} p_{\tilde{\gamma }}\left(x\right) & =\kappa^{\kappa }\frac{{\left(x\right)}^{\kappa -1}}{\Gamma (\kappa )}\exp\left(-\kappa x\right)\times \frac{1}{\sqrt{2\pi }\sigma_{Z}x}\exp\left(-\frac{{\left(\ln\left(x\right)-\eta_{Z}\right)}^{2}}{2\sigma_{Z}^{2}}\right), \end{array}$$

### 4.3. Packet Success Probability

By leveraging on the approximation of the statistical properties of the SINR, the packet success probability can be expressed as:

(13) $$\begin{array}{c} p_{i,j}=1-\int_{0}^{\infty }PER\left(x\right)p_{\tilde{\gamma }}\left(x\right)dx \end{array}$$

The term PER(x) represents the packet error rate (PER) as a function of SINR, which may have different expressions depending on the specific system parameter settings. Here, the PER(x) is characterized using the model explained in [29], and calculated adopting LUTs that report the block error rate (BLER) as a function of the SNR for a given packet size, MCS, scenario (highway or urban), and a reference speed between transmitter and receiver with no re-transmissions. The LUTs are used to map the SINR to the packet error rate, assuming that the negative effect of the interference on the received signal is equivalent to additional noise. Without active interfering vehicles, the SINR reduces to the SNR. We remark that the derivation of an exact expression of PER(x) is a cumbersome task, and these LUTs are widely used in the related literature (e.g., [21]).

## 5. Results and Discussion

In this section, we present comprehensive results to verify and compare the performance of NR-V2X Mode 2 with the LTE-V2X Mode 4 communication protocol in a platooning scenario. A variety of propagation scenarios and MAC configurations are tested to validate the analytical model and the proposed approximation. The shadowing components in the propagation environment are identically distributed. Additionally, we refer to the shadowing spatial correlation model from [37], where the decorrelation distance in urban settings is equal to 10 m. Therefore, the shadowing components are practically uncorrelated in the platooning scenario under consideration, where the distance between vehicles is greater than 13 m.

The packet generation process is implemented by enforcing the model introduced in [37], which represents an abstraction of the packet exchange dynamics specifically tailored for vehicle networks. Depending on the outcome of the SB-SPS selection, the approximated SINR is calculated for each generated packet, and the PER is determined by sampling the BLER-SINR curves that were reproduced from [29] for LTE-V2X and [38] for NR-V2X, respectively.

In our investigation, we consider a single 10 MHz channel reserved specifically for V2V communications. Additionally, we assume perfect synchronization, and we set the transmission power $P_{tx,i}$ to 30 dBm for each vehicle. All the other relevant parameters considered for the evaluation are listed in Table 2 and the considered values are set to be compliant with the 3GPP specifications.

To verify the accuracy of the proposed approximation rationale as described in Section 4, extensive Monte Carlo simulations using MatLab are also performed for both NR-V2X and LTE-V2X, generating $10^{6}$ independent channel realizations for each vehicle and packet transmission attempt. Performance is evaluated in terms of packet success probability by changing the number of interfering vehicles, the number of resources (i.e., the number of transport blocks in a selection window), and the separation between the transmitter and receiver. At each data point for the simulations, the standard deviation is also displayed as a vertical error bar.

### 5.1. Impact of Interfering Vehicles

**Figure 3:**
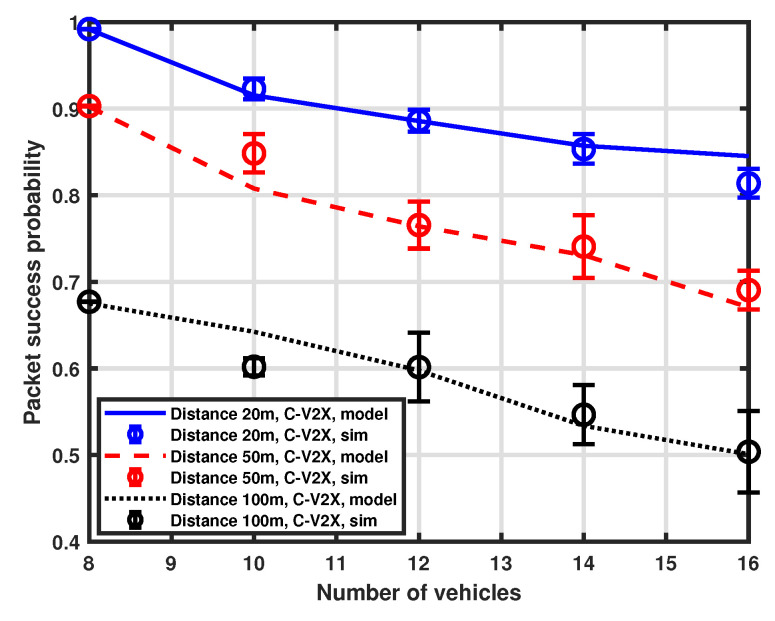
Packet success probability as a function of the number of vehicles.

**Figure 4:**
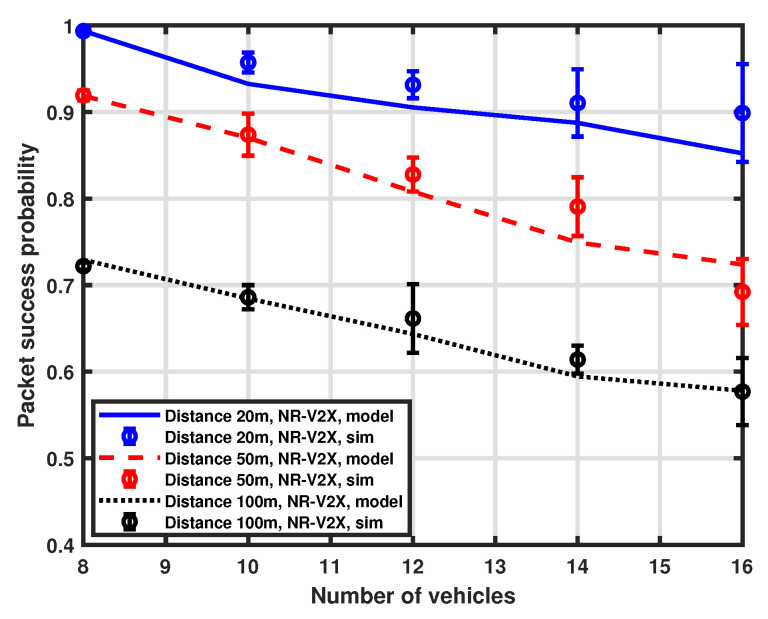
Packet success probability as a function of the number of vehicles.

As a first investigation, we consider packet success probability as a function of the number of interfering vehicles in the platoon. In this case, the total number of resources in the selection window is set to 8 and the number of vehicles ranges from 8 to 16. Moreover, different distances (i.e., 20 m, 50 m, and 100 m) between the transmitter (the tagged vehicle) and the receiver (the platoon head) are considered. A comparison between the results obtained with our approximation procedure and simulations is shown in Figure 3 for the LTE-V2X and Figure 4 for the NR-V2X. It is possible to observe that the developed MMA approximation provides good accuracy for all the considered system parameter settings.

**Figure 5:**
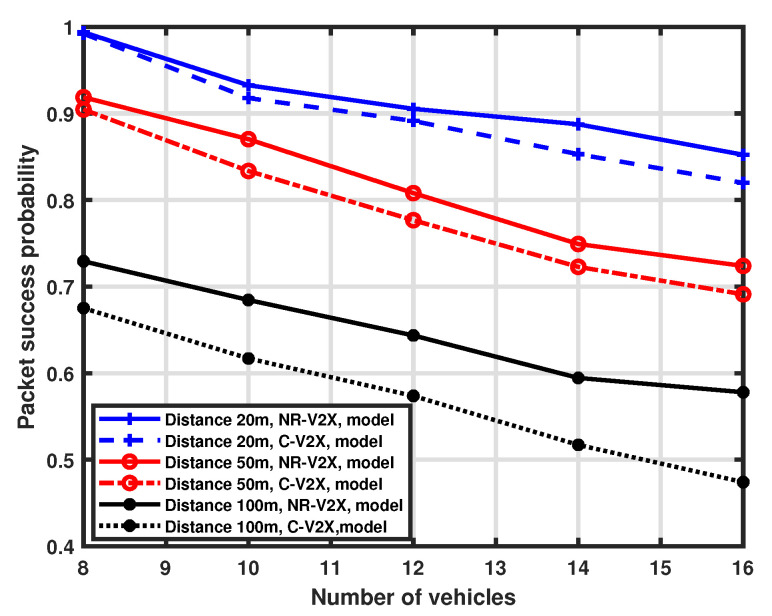
Packet success probability as a function of the number of vehicles.

Figure 5 provides a comparison between the packet success probability of LTE-V2X and NR-V2X. We can observe that the performance of the considered architecture is similar when the distance is set to 20 m and 50 m, with NR-V2X exhibiting a slight performance improvement for LTE-V2X. However, when the distance is increased to 100 m, the performance gap increases, showing a non-negligible performance improvement of NR-V2X over LTE-based systems. This may be due to the interplay of carrier sensing in SB-SPS and the different physical access parameters for NR-V2X in contrast to LTE-V2X. NR-V2X can more flexibly react to slot collisions due to hidden interfering vehicles at a larger distance with the semi-persistent slot allocation scheme.

Moreover, the chance of successfully receiving packets decreases as the number of interfering vehicles increases, as expected. Nevertheless, the slope of performance degradation is not very pronounced, thus demonstrating the effectiveness of the SPS scheme in allocating resources among vehicles.

### 5.2. Impact of Available Resources

**Figure 6:**
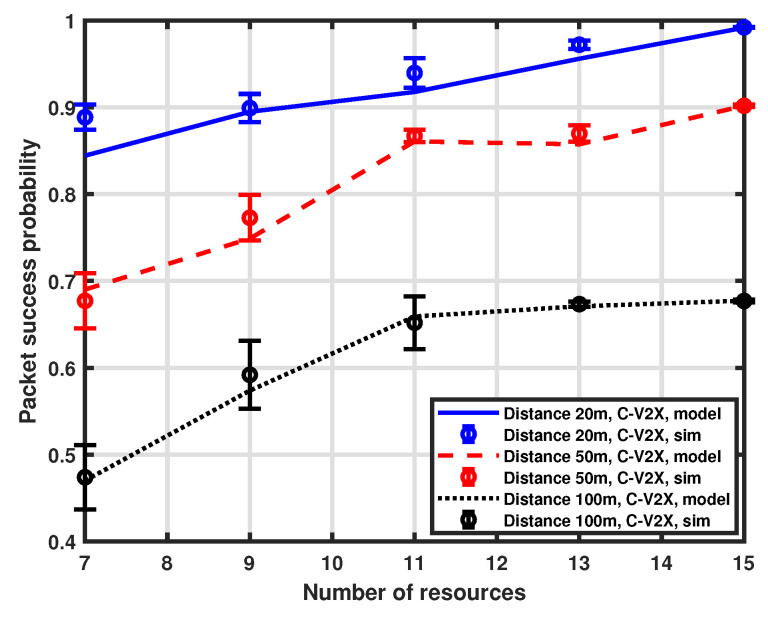
Packet success probability as a function of the number of resources.

**Figure 7:**
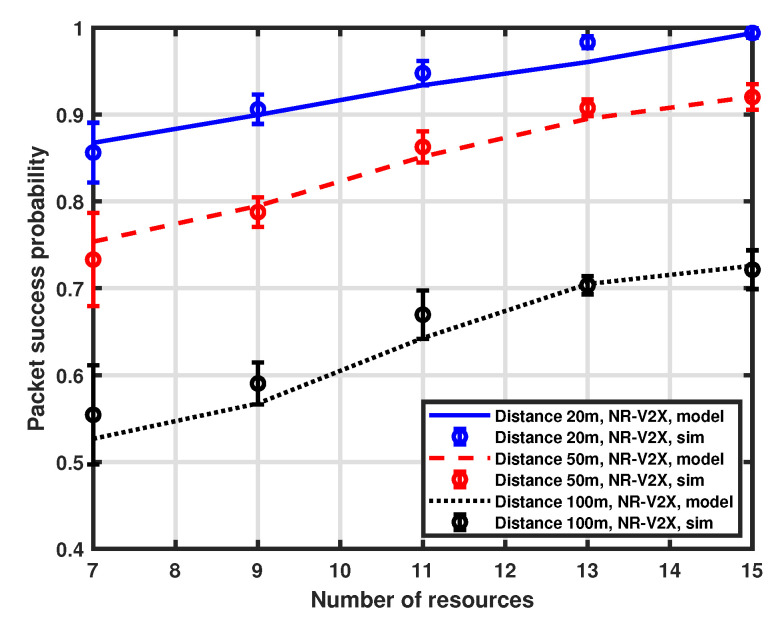
Packet success probability as a function of the number of resources.

We also investigate the influence of the available resources on packet success probability. Both LTE-V2X and NR-V2X use the same number of resources for a fair comparison, as the purpose is the comparison of the ability to reject multiple access interference. However, the availability of allocable resources in a real-world case could be higher for NR-V2X due to the different options for bandwidth and MAT. For this analysis, we again consider three distinct distances between the transmitter and the receiver (i.e., 20 m, 50 m, and 100 m). The number of interfering vehicles is 15 and the number of available resources in the selection window ranges from 7 to 15. The validation of the proposed approximation is reported in Figure 6 and Figure 7, which show the performance of LTE-V2X and NR-V2X, respectively, and again demonstrates a good accuracy of the MMA approximation.

**Figure 8:**
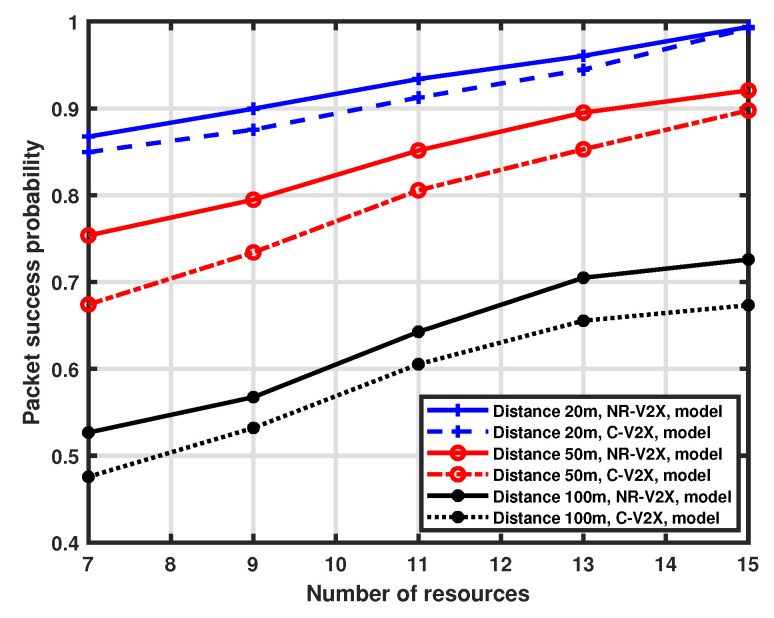
Packet success probability as a function of the number of resources.

The performance of the NR-V2X is also compared with that of the LTE-V2X in Figure 8. As expected, the probability of success increases as the number of available resources increases. This is because, for a fixed number of interfering vehicles, the increase in available communication slots in the selection window implies a reduced impact of interference. It is to be noted that, when the number of resources is equal to the number of vehicles (i.e., 15), each vehicle has the option to reserve a dedicated communication slot, so interference does not affect the performance, which is mainly determined by the distance between the tagged vehicle and the platoon head. Finally, we observe that the NR-V2X provides a higher success probability than LTE-V2X.

### 5.3. Impact of the Distance between Transmitter and Receiver

**Figure 9:**
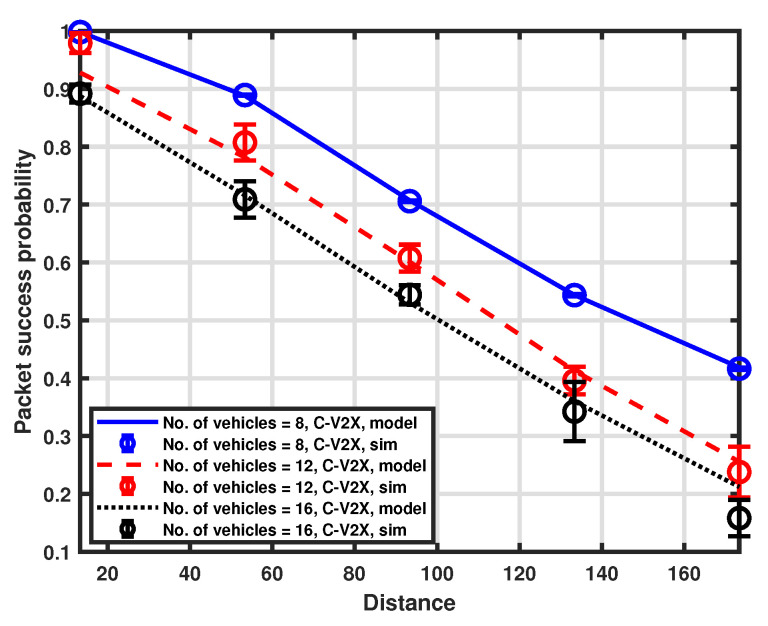
Packet success probability as a function of distance.

**Figure 10:**
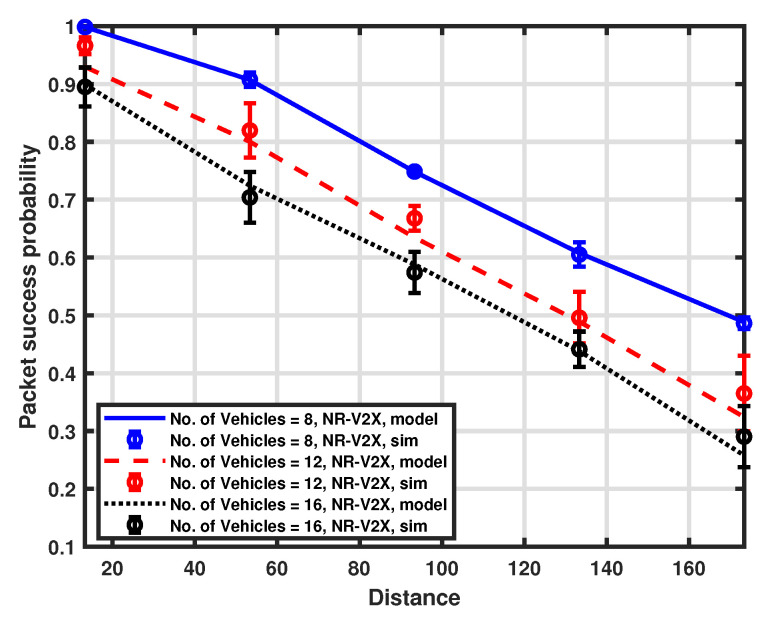
Packet success probability as a function of distance.

**Figure 11:**
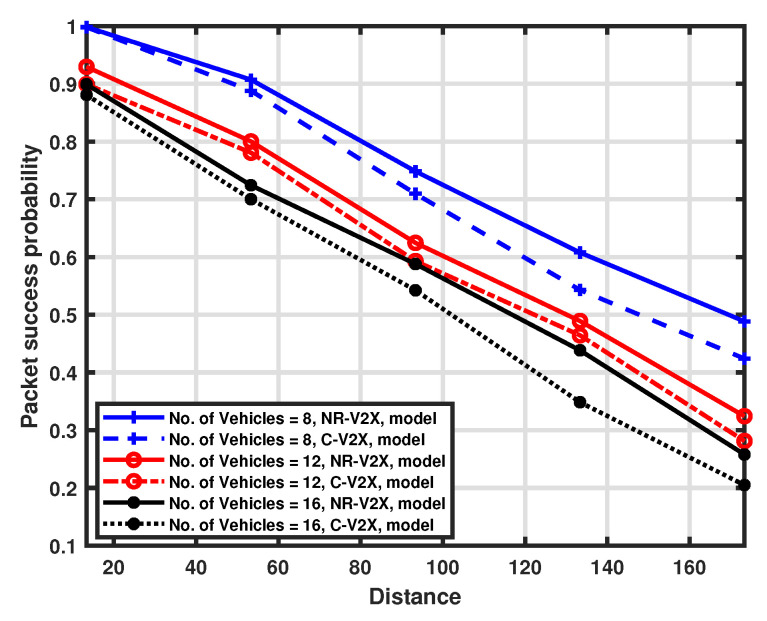
Packet success probability as a function of distance.

The packet success probability is also examined for the separation of the distance between the transmitter and receiver for a fixed number of resources in the selection window, which is set to 8. Different platoon sizes are considered by setting the number of vehicles to 8, 12, and 16, respectively. The validation of the proposed approximation model is illustrated in Figure 9 and Figure 10, while the comparison between LTE-V2X and NR-V2X is shown in Figure 11.

The probability of success decreases by increasing the distance between the transmitting vehicle and the platoon head. This is primarily caused by the impact of high attenuation, which worsens the received SNR, and the reduced carrier sensing efficiency, which induces a higher packet error rate. Because of the higher interference level, we can see that performance deterioration is more pronounced when the number of communicating vehicles is higher than the number of resources. We can also deduce that the impact of channel sensing failures may have a greater influence on performance than an increase in the number of vehicles. Indeed, the performance degradation observed when switching from a platoon size of 12 to a platoon size of 16 is very low if compared to a change in distance. Finally, from Figure 11, we can appreciate the performance comparison between the two considered communication modes, and NR-V2X is shown to provide better performance than LTE-V2X.

## 6. Conclusions

Vehicle platooning is a prominent use case and a challenging scenario for vehicular communications. Several research studies have investigated the performance of cellular sidelink communications based on LTE-V2X Mode 4, but peculiar aspects such as multiple access interference in vehicle platooning scenarios have not yet been investigated. In this context, the resource allocation in sidelink operations in C-V2X has a major impact on the performance of the sidelink communication in real-world use-case scenarios. NR-V2X specifically addresses the requirements of platooning scenarios to overcome the limitations of LTE-V2X.

In this work, we presented a comparative study of LTE-V2X and NR-V2X sidelink communications in a vehicle platooning scenario. We established and verified an analytical approach to estimate the performance of LTE-V2X Mode 4 and 5G-NR Mode 2 communications based on available resources, the number of interfering vehicles, and their relative positions. The moment matching approximation (MMA) method was enforced to approximate the statistics of the signal-to-interference-plus-noise ratio and allowed us to derive the packet success probability accounting for realistic propagation impairments described by a composite channel model.

The comparison of the two technologies confirmed that the proposed approach provides a concise and accurate approximation of the attainable performance, confirming the superior performance with NR-V2X against LTE-V2X, particularly for high inter-vehicle distance and a large number of vehicles, thus enabling our framework to be effectively used for optimization and testing of the resource allocation for sidelink communications in 5G-V2X.

In terms of future work, we intend to: (i) design a platform for both Mode 2 and Mode 4, where vehicles can switch between the two modes as needed and can select and modify the ideal RRI value based on their real-time vehicle density; (ii) expand our framework by incorporating a comprehensive analytical model of the physical access; and (iii) use experimental campaigns to strengthen the validation of the V2X analytical model.

## Figures and Tables

**Table 1:** Comparison of LTE-V2X and NR-V2X.

Features	LTE-V2X	NR-V2X
Communication types	Broadcast	Broadcast, Groupcast & Unicast
MCS	QPSK & 16-QAM	QPSK, 16-QAM & 64-QAM
Waveform	SC-FDMA	OFDM
Re-transmissions	Blind	HARQ
PHY channels	PSCCH & PSSCH	PSCCH, PSSCH & PSFCH
Control and data multiplexing	FDM	TDM
DMRS	Four/sub-frame	Flexible
Sub-carrier spacing	15 kHz	sub-6 GHz: 15, 30, 60 kHz & mmWave: 60, 120 kHz
Scheduling interval	one sub-frame	slot, mini-slot or multi-slot
Sidelink modes	Mode 3 & Mode 4	Mode 1 & Mode 2
Sidelink sub-modes	N/A	Modes 2(a) & 2(d)

**Table 2:** System parameters.

Parameter	Value (or Range)
Transmission power $\left(P_{t}\right)$	30 dBm
Reference path gain $\left(c_{0}\right)$	−42.5 dB
Path loss exponent (α)	3.68
Noise power $\left(\sigma_{w}^{2}\right)$	−95 dBm
Channel bandwidth (W)	10 MHz
Fading spread $\left(\sigma_{i}\right)$	3
Number of resources in selection window (N)	up to 15
Number of vehicles (M)	up to 16
Distance $d_{i,j}$	[10–200] m
Channel sensing time $\left(T_{s}\right)$	LTE-V2X: 1 s; NR-V2X: 0.5 s
Power level threshold $(P_{th)}$	[−128, −2] dBm
Probability of keeping the resources $\left(p_{k}\right)$	[0 80%]

## Data Availability

The data presented in this study are available on request from the corresponding author.
